# ZGRF1 promotes end resection of DNA homologous recombination via forming complex with BRCA1/EXO1

**DOI:** 10.1038/s41420-021-00633-7

**Published:** 2021-09-22

**Authors:** Shuang Yan, Man Song, Jie Ping, Shu-ting Lai, Xiao-yu Cao, Chen-Jun Bai, Da-Fei Xie, Hua Guan, Shan-shan Gao, Ping-Kun Zhou

**Affiliations:** 1 Institute for Environmental Medicine and Radiation Hygiene, School of Public Health, University of South China, Hengyang, Hunan Province People’s Republic of China; 2 Department of Radiation Biology, Beijing Key Laboratory for Radiobiology, Beijing Institute of Radiation Medicine, Beijing, People’s Republic of China; 3 State Key Laboratory of Proteomics, National Center for Protein Sciences, Beijing Institute of Radiation Medicine, Beijing, People’s Republic of China; 4 College of Life Sciences, Hebei University, Baoding, He Bei Province People’s Republic of China

**Keywords:** Targeted therapies, DNA

## Abstract

To maintain genomic stability, the mammalian cells has evolved a coordinated response to DNA damage, including activation of DNA repair and cell cycle checkpoint processes. Exonuclease 1 (EXO1)-dependent excision of DNA ends is important for the initiation of homologous recombination (HR) repair of DNA breaks, which is thought to play a key role in activating the ATR-CHK1 pathway to induce G2/M cell cycle arrest. But the mechanism is still not fully understood. Here, we report that ZGRF1 forms complexes with EXO1 as well as other repair proteins and promotes DNA repair through HR. ZGRF1 is recruited to DNA damage sites in a MDC1-RNF8-BRCA1 dependent manner. Furthermore, ZGRF1 is important for the recruitment of RPA2 to DNA damage sites and the following ATR-CHK1 mediated G2/M checkpoint in response to irradiation. ZGRF1 null cells show increased sensitivity to many DNA-damaging agents, especially PARPi and irradiation. Collectively,our findings identify ZGRF1 as a novel regulator of DNA end resection and G2/M checkpoint. ZGRF1 is a potential target of radiation and PARPi cancer therapy.

## Introduction

DNA double-strand break (DSB) is the most fatal type of DNA damage. Failure to properly repair DSB can lead to chromosomal aberrations,genome instability and overall increase in cell death [1, 2]. In mammalian cells, there are two prominent repair pathways that repair double strand breaks (DSBs): homologous recombination (HR) repair and non-homologous end-joining (NHEJ) mechanisms [3–5]. In NHEJ pathway, the break ends are directly ligated without homologous templates [6]. So, NHEJ is an error-prone repair, commonly associated with the presence of insertions and deletions at DSBs [4, 7]. HR is different from NHEJ, which needs an intact homologous template, and primarily functions in the S/G2 phases [8, 9].

A key step in HR repair is DNA end resection, which is initiated by the human MRE11-RAD50-NBS1 (MRN) complex with CtIP to generate a short 3′ single-stranded DNA (ssDNA) tail [8–10]. The MRN complex first binds to the DNA double-strand break end [11, 12], and then the nuclease CtIP is phosphorylated by CDK and also binds to the break end,stimulating DNA strand processing in the 5′→3′ direction [13–15]. MRN and CtIP cooperate to produce a 50–100 nucleotide 3′-OH overhang single strand DNA (ssDNA) [16, 17]. In this process, ATM stimulates the activity of CtIP and MRE11 [18–20]. 3′ssDNA is coated with RPA (replication protein A), which becomes nuclease degradation and removes secondary structure; then, mediated by BRCA2 protein, RPA is replaced by recombinase RAD51 [21]. RAD51 mediates the invasion of the DNA double-stranded template and the following complete the repair process [22].

Exonuclease 1 (EXO1) is an important nuclease involved in the DNA repair system that helps to maintain genomic stability, to modulate DNA recombination, and to mediate cell cycle arrest. In the process of DNA end excision, EXO1 acts as a 5′→3′ excision nuclease [23–25], and BLM and DNA2 interact functionally and physically to form a core complex, which in the presence of human RPA moves along DNA is excised in the 5′→3′ direction, and the MRN complex recruits EXO1 to DNA and improves its synthesis ability [26–29]. Although EXO1 can cut DNA ends by itself, BLM makes EXO1 a more effective nuclease, which can excise thousands of nucleotides at the ends of DNA [27, 30]. BLM is a member of the RecQ family of helicases which interacts with EXO1 and unwinds DNA in mammals cells [31]. It has been reported EXO1 participates in the formation of ssDNA and the following activation of ATR-CHK1 checkpoint in response to DNA damage [32]. Studies also have shown that the depletion of BLM only slightly impairs DSB resection and subsequent ATR-mediated signaling, indicating that there may be other resection processes action factor [33].

To ensure accurate segregation of chromosomes,cells must prevent entry into mitosis in the presence of DNA damage. The G2/M checkpoint plays an essential role in preventing cells from entering mitosis and providing an opportunity for repair when DNA is damaged [34]. RPA2 polymerizes on this ssDNA to generate a platform that also activates the central signaling pathway orchestrating DNA replication responses, the ATR pathway [34–36]. RPA-ssDNA complex recruits the ATR/ATRIP complex through direct interaction with ATRIP to localize it to the fork [34, 37]. The localization of ATR/ATRIP complex to DSBs sites sets in motion of the ATR signaling cascade, which results in the phosphorylation of CHK1 S345 and RPA2 S4S8 and the initiation of G2/M checkpoint [37].

Human ZGRF1 (zinc finger GRF-type containing 1, C4orf21) protein shares homology with Saccharomyces cerevisiae Mte1/Dbl2 (Mph1-associated telomere maintenance protein 1) in its N-terminal DUF2439 domain [38]. The C terminus of ZGRF1 gene is not conserved in Mte1/Dbl2 and encodes a Zn finger DNA binding domain and a helicase domain [39]. Human ZGRF1 is largely uncharacterized, but genome-wide small interfering RNA (siRNA) knockdown screens have suggested a role for ZGRF1 as a regulator of HR and ICL (interstrand crosslink) repair [39]. ZGRF1 has the activity of 5’ to 3’ DNA helicase that promotes the resolution of replication-blocking DNA lesions in HR pathway [39]. It also has been reported ZGRF1 is important for cell division,and ZGRF1 mutant cells show obvious mitotic defects. Those remind us ZGRF1 may be associated with the G2/M checkpoint in response to DNA damage. In addition,ZGRF1 was reported to be recruited to sites of DNA damage and promote HR by directly stimulating the RAD51 recombinase activity [39]. However, the mechanism of ZGRF1 recruitment to DNA damage sites and the connection of ZGRF1 with other HR assciated proteins such as BRCA1, EXO1 remains unclear.

Here,we report that ZGRF1 regulates DNA repair and cells survival upon DSB induction. We demonstrate that ZGRF1 forms complexes with multiple HR repair proteins including EXO1 and BRCA1, and is recruited to DNA damage sites in a MDC1-RNF8-BRCA1 dependent manner. ZGRF1 is important for efficiency of HR in DSBs in human cells by affecting EXO1-mediated DNA-end resection. Significantly, knock out of ZGRF1 impairs the recruitment of RPA2 to DSB sites, and considerably reduces CHK1, RPA2 phosphorylation and the initiation of G2/M checkpoint. These results collectively demonstrate an important role of ZGRF1 in DSBs-induced G2/M cell cycle arrest by regulating EXO1-mediated DSB end resection. Furthermore, higher expression levels of ZGRF1 predicts poor prognosis in cancer therapy, and ZGRF1 knock-out cancer cells are highly sensitivity to DNA damage stress and PARPi. All together,we provide a new insight into the molecular mechanisms that regulate the HR DNA end resection and the potential target for tumor therapy.

## Results

### ZGRF1 interacts with DNA damage response proteins and localizes to sites of DNA Damage

**Fig. 1:**
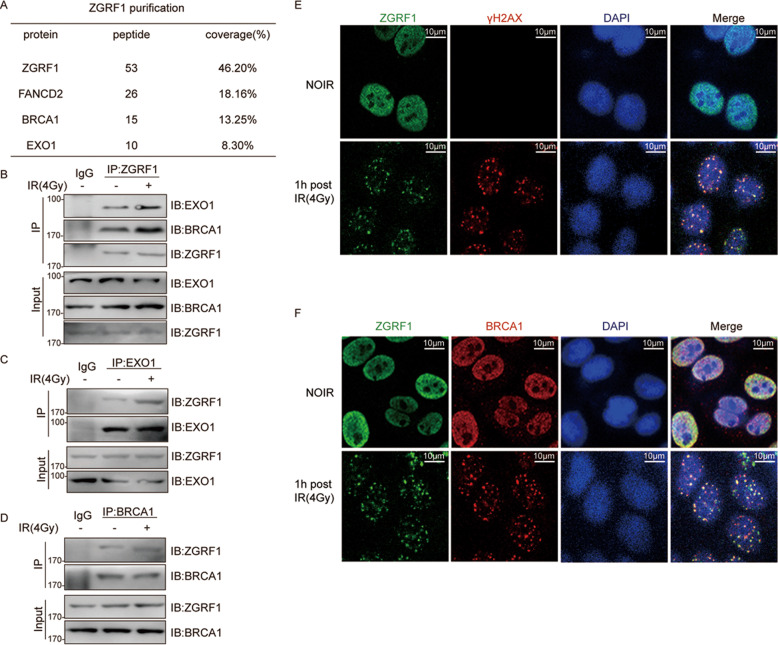
ZGRF1 interacts with DNA damage associated proteins and localizes to sites of DNA Damage. **A** HeLa cells were treated with 4 Gy irrdiation, then Co-IP was performed using ZGRF1 antibody, mass spectrometry was used to identify ZGRF1-associated proteins. **B**–**D** Co-immunoprecipitation (Co-IP) assays were performed using the extracts of HeLa cell lines with the indicated antibodies to confirm the interaction between ZGRF1 and EXO1, BRCA1. IP samples were separated by SDS–PAGE and immunoblotted for the indicated proteins. **E**, **F** One hour after 4 Gy irradiation, HeLa cells were harvested and immunostained with the indicated antibodies.

To investigate the role of ZGRF1 in DNA repair, HEK-293T cells transfected Flag-tagged ZGRF1, ZGRF1 purification was performed by Co-IP with Flag beads after ionizing radiation (IR) treatment,chromatin-associated ZGRF1 complexes were isolated and subjected to mass spectrometry analysis. We identified several DNA repair proteins, including BRCA1 and EXO1 (Fig. 1A). The association of DNA repair proteins suggested a possible role of ZGRF1 in the regulation of DNA repair. To confirm this interaction, we performed immunoprecipitation assay with ZGRF1 antibody using chromatin-free cell extracts from HeLa cells. The western blotting results showed that ZGRF1 interacted with EXO1 and BRCA1 both before and after irradiation. And interestingly, the interaction between ZGRF1 and BRCA1, EXO1 was increased in response to IR induced DNA damage (Fig. 1B). To further investigate the interaction, Co-IP assay was performed using EXO1 (Fig. 1C) and BRCA1 (Fig. 1D) antibodies, the results also showed ZGRF1 interacted with both EXO1 and BRCA1. And the interaction was increased after DNA damage induction. These results collectively demonstrated that ZGRF1 forms a complex with DNA repair proteins including EXO1 and BRCA1 in a manner independent of chromatin. To study whether ZGRF1 is involved in DNA damage response, immunostainning assay were performed using HeLa cells 1 h after 4 Gy irradiation, the data showed ZGRF1 colocalizated with γH2AX and BRCA1 (Fig. 1E, F). It indicates ZGRF1 is recruited to sites of DNA damage after irradiation.

### ZGRF1 participates in the HR repair pathway

**Fig. 2:**
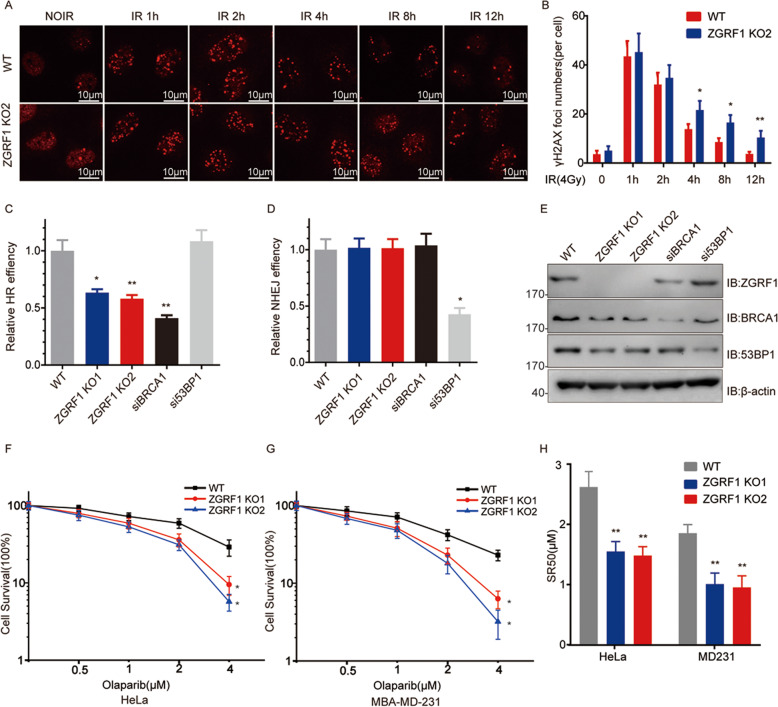
ZGRF1 deletion impairs HR repair. **A** ZGRF1 depletion inhibits the efficiency of DNA damage repair as shown by the increased residual γH2AX foci in HeLa cells. **B** Quantification of γH2AX foci in the HeLa cells at different time after 4 Gy irradiation. Data are means ± SD from three independent experiments (50 cells were scored in each experiment). *P < 0.05; **P ≤ 0.01, two-tailed Student’s *t* test. **C** HR efficiency was determined using the direct repeat GFP (DR-GFP) reporter assay. **D** The NHEJ efficiency was determined using the EJ5-GFP reporter assay. BRCA1 and 53BP1 siRNAs were used as a positive or negative control, respectively. Data are means ± SD from three independent experiments. *P < 0.05; **P ≤ 0.01. two-tailed Student’s *t*-test. **E** Knocking down efficiency of the indicated siRNA were detected by western blotting. **F**–**H** ZGRF1 deletion sensitizes HeLa (**F**) and MD231 (**G**) cancer cells to the PARP inhibitor olaparib, measured by colony formation assay. SR50 (**H**) represents the concentration for 50% cell survival. Data are means ± SEM from three independent experiments. *P < 0.05; **P ≤ 0.01. Two-way analysis of variance (ANOVA).

To study the function of ZGRF1 in DNA damage repair, ZGRF1 knock out HeLa and MD231 cells were built by using CRISPR/Cas9 system. Given that ZGRF1 forms a complex with DNA repair proteins and localizes to DNA damage sites,we postulated that ZGRF1 plays a role in DNA damage repair. To detected the efficiency of DNA repair, a typical marker of DNA DSB, γH2AX foci formation was examined in wild-type (WT) and ZGRF1 knock-out HeLa cells. As shown in Fig. 2A, B, as compared with WT cells, ZGRF1-deleted cells resulted in elevated levels of residual γH2AX foci 4 h or longer after 4 Gy irradiation, suggesting that ZGRF1 is necessary for the efficiency of DNA DSB repair. To find out which pathway of DSB repair ZGRF1 involves in, HR and NHEJ assays were conducted using DR-GFP and EJ-GFP system in ZGRF1 WT and deletion HeLa cells. The results showed ZGRF1 deletion reduced HR repair efficiency by 40% (Fig. 2C), but did not effect NHEJ efficiency (Fig. 2D). The knock-down expression of BRCA1 or 53BP1 siRNA were tested by western blotting (Fig. 2E). Those data are coordinated with the binding of ZGRF1 and the HR associated proteins EXO1, BRCA1.

PARP inhibitors, such as Olaparib, AZD2281 and Niraparib, have been designed and tested for many years and became new class of chemotherapeutic agents directed at targeting cancers with BRCA mutations and HR defect. Searching for new biomarkers that can efficiently identify tumors that are sensitive to PARP inhibitor treatment would widen the prospective patient population benefit from PARPi. The cancer cells with deleted ZGRF1, which impairs HR repair, may also be sensitive to PARP inhibitor. To investigate the role of ZGRF1 in PARPi response, the colony formation assays were performed in WT and ZGRF1 knock out HeLa (Fig. 2F) and MD231 (Fig. 2G) cancer cells. The results showed ZGRF1 deletion significantly sensitized both HeLa and MD231 cells to Olaparib (Fig. 2F, G). The SR50 concentration of ZGRF1 deleted HeLa and MD231 to Olaparib reduced 40% and 50%, respectively, compared to WT (Fig. 2H). It indirectly also suggests an important role of ZGRF1 in the HR pathway.

### Recruitment of ZGRF1 to DNA damage sites depends on BRCA1 and MDC1-RNF8 pathway

**Fig. 3:**
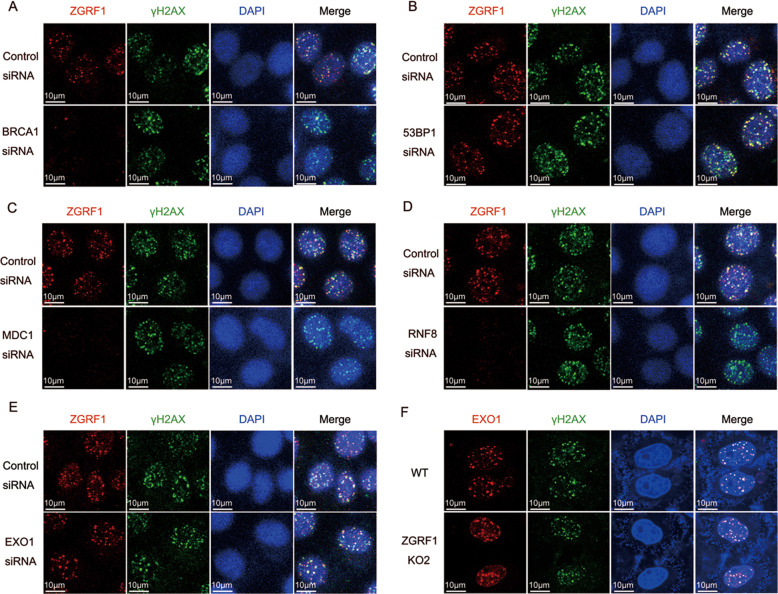
ZGRF1 localization to DNA damage sites depends on BRCA1 and MDC1-RNF8 pathway. **A**, **B** ZGRF1 accumulation at DSB sites requires BRCA1 but not 53BP1. HeLa cells transfected with control or BRCA1 or 53BP1 siRNA were treated with IR (4 Gy) treatment and 1-h recovery, then cells were harvested and immunostained with ZGRF1 and γH2AX antibodies. **C**, **D** Knock-down expression of MDC1 or RNF8 abolished ZGRF1 accumulation at DSB sites. ZGRF1 and H2AX foci formation was examined in HeLa cells transfected with control or MDC1 or RNF8 siRNA were treated with IR (4 Gy) treatment and 1-h recovery. **E** ZGRF1 acumulation at DSB sites does not require EXO1. HeLa cells transfected with control or EXO1 siRNA were treated with IR (4 Gy) treatment and 1-h recovery,then cells were harvested and immunostained with ZGRF1 and γH2AX antibodies. **F** EXO1 accumulation at DSB sites does not require EXO1. HeLa cells were were treated with IR (4 Gy) treatment and 1-h recovery, then cells were harvested and immunostained with EXO1 and γH2AX antibodies.

To investigate the mechanism of how ZGRF1 is recruited to damage sites,we examined the localization of ZGRF1 at DSB sites in cells with down-regulated early DNA repair factors including BRCA1 and 53BP1 by siRNA. The result showed depletion of BRCA1 significantly reduced the recruitment of ZGRF1 to DSB sites (Fig. 3A). And depletion of 53BP1 had no effects on the recruitment of ZGRF1 to damage sites (Fig. 3B). It is also in accord with our result that ZGRF1 was involved in HR pathway, but not NHEJ.

Since MDC1-RNF8 pathway regulates the localization of BRCA1 to DSB sites, we went further to investigate whether MDC1 regulates the recruitment of ZGRF1 to DSB sites. Markedly,we found that the localization of ZGRF1 to DSB sites was also impaired in the cells with MDC1 depletion (Fig. 3C). To further confirm the role of MDC1-RNF8 pathway in ZGRF1 recruitment, we investigated the recruitment of ZGRF1 to DSB sites in RNF8 deleted cells. Strikingly, the recruitment of ZGRF1 to DSB sites was significantly impaired in RNF8 deletion cells (Fig. 3D). Taken together, our data strongly suggest that MDC1-mediated pathway is involved in the recruitment of ZGRF1 to DSB sites.

Since BRCA1 is essential for both ZGRF1 and EXO1 localization to sites of DNA damage, we wonder whether ZGRF1 regulates EXO1 recruitment to DNA damage sites. However, deletion of ZGRF1 did not affect EXO1 foci formation. Then, we knocked down EXO1 using siRNA, and tested ZGRF1 foci, the results showed EXO1 did not affect ZGRF1 foci formation, either (Fig. 3E and F). It indicates that ZGRF1 possibly regulates DNA end resection through its helicase activity to promote EXO1 mediated DNA end resection.

### ZGRF1 regulates DNA end resection

**Fig. 4:**
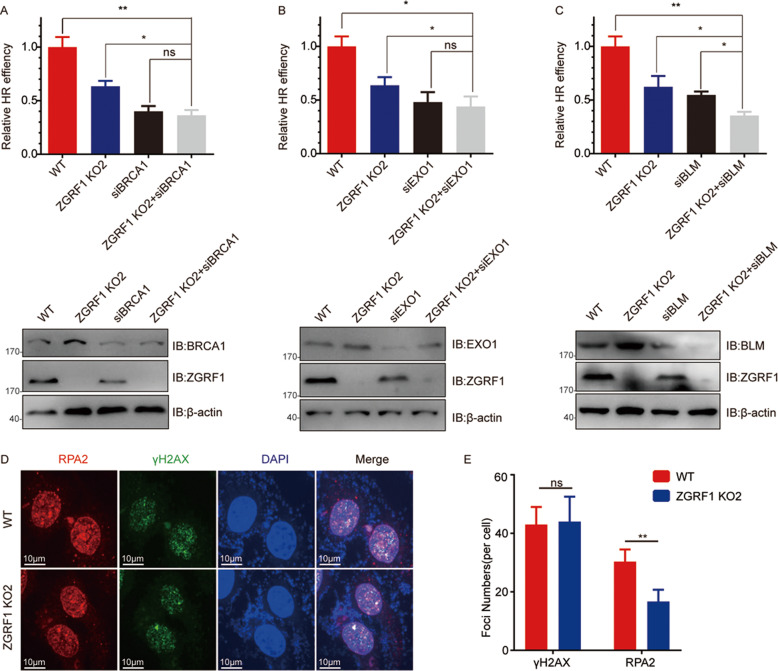
ZGRF1, BRCA1 and EXO1 work in the same pathway to regulate DNA end resection. **A**–**C** HR efficiency was determined using the DR-GFP reporter assay. Data are means ± SD from three independent experiments. *P < 0.05; **P ≤ 0.01. two-tailed Student’s *t*-test. **D** RPA2 accumulation at sites of 4Gy–induced DNA damage. One hour after irradiation, HeLa cells were harvested and immunostained with γH2AX and RPA2 antibodies. **E** Quantification of γH2AX and RPA foci in the cells 1 h after 4 Gy irradiation. Data are means ± SD from three independent experiments (50 cells in each experiment). *P < 0.05; **P ≤ 0.01.two-tailed Student’s *t*-test.

Given that ZGRF1 forms complex with BRCA1 and EXO1, and both ZGRF1 and EXO1 are in the downstream of BRCA1 in HR repair, it perhaps they function in the same pathway. To test this hypothesis, the HR repair assay were performed. As shown in Fig. 4A, double knockdown of ZGRF1 and BRCA1 showed similar phenotype as either single knockdown or deletion. And double knockdown of ZGRF1 and EXO1 showed similar phenotype as either single knockdown or deletion as well. These results clearly indicate that ZGRF1 and BRCA1, EXO1 function in the same pathway.

As ZGRF1 is a 5′- to −3′ DNA helicase,and it has been reported BLM regulates the EXO1-mediated DNA end resection through its helicase activity.We wonder whether ZGRF1 works together with BLM to promotes the EXO1-mediated DNA end resection of IR-induced DSBs.To answer this question,BLM were knocked down in ZGRF1 deletion cell lines and then HR assay was performed,the result showed double knockdown ZGRF1 and BLM reduced the HR efficiency compared with either single knockdown or deletion (Fig. 4C). This indicateds ZGRF1 and BLM function in different pathways.Then we detected the effects of ZGRF1 deletion in RPA2 foci formation, the marked of DNA end resection. As shown in Fig. 4D and E, RPA2 foci formation was significantly decreased in ZGRF1 deletion cells. Out results strongly indicates ZGRF1 promotes the DNA end resection.

### ZGRF1 regulates G2/M checkpoint

**Fig. 5:**
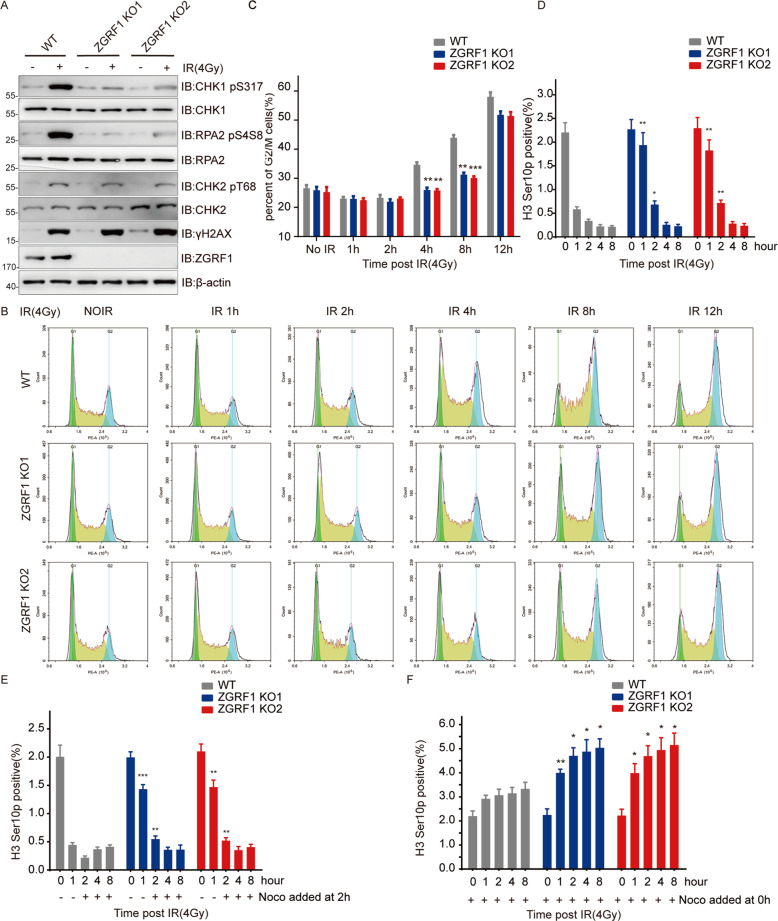
ZGRF1 regulates G2/M checkpoint. ZGRF1 is required for efficient ATR activation in response to IR treatment. HeLa cells were harvested following IR (4 Gy) treatment and a 1-h recovery, and immunoblotted for the indicated proteins. **B** Flow cytometric histograms of cell cycle detection. The WT and ZGRF1 deletion HeLa cells were treated with 4-Gy irradiation. Cell cycle was detected at indicated time points after treatments. **C** Quantification of percentage of G2/M cells. Data are means ± SD from three independent experiments. *P ≤ 0.05; **P ≤ 0.01. **D**–**F** HeLa cells were fixed at the indicated time points after IR. Nocodazole (Noco) was added 2 h (**E**) or 0 h (**F**) after IR. The bar chart shows the percentage of Histone H3-Ser10p–positive cells. H3-Ser10p–positive percentage of ZGRF1 deletion group was compared with WT group. *P < 0.05. Error bars represent SDs.

The generation of RPA-coated ssDNA is also essential for the activation of ATR-CHK1 pathway under genomic pressure. Indeed, ZGRF1 deletion reduced IR-induced CHK1 and RPA2 phosphorylation, but had no effect on the total level of CHK1 or RPA2 (Fig. 5A). Since CHK1 phosphorylation plays a key role in the regulation of G2/M checkpoints, we next investigated the role of ZGRF1 in cell cycle checkpoints. As shown in Fig. 5B and C, the percent of G2/M cells was significantly decreased at 4 h and 8 h after irradiation in ZGRF1 deletion cells, compared with the WT. And 12 h after irradiation, the percent of G2/M cells shown no difference between WT and ZGRF1 deletion cells. The G2/M checkpoint arrestment in response to DNA damage was delayed by ZGRF1 deletion.

To test how ZGRF1 effects the G2/M checkpoint arrestment,the fraction of mitotic cells were detected by flow cytometer using phospho-histone 3 Ser10p antibody,a maker of mitotic cells. In response to irradiation, the fraction of mitotic cells in WT cells was significantly reduced 1 h after irradiation treatment (Fig. 5D). However, the ZGRF1 deletion showed more mitotic cells at 1 h and 2 h after irradiation compared with WT. However, the percent of mitotic cells showed no difference at both 4 h and 8 h after irradiation in WT and ZGRF1 deletion cells. It implies that ZGRF1 deletion impairs the initiation of G2/M checkpoint but not maintenance.

To further investigate this hypothesis, cells were treated with 1 μm nocodazole at 0 h (Fig. 5F) or 2 h (Fig. 5E) after irradiation to inhibit cells from going out of the mitotic phase,then were immunostained with H3 Ser10p antibody labeled and tested by flow cytometer. As shown in Fig. 5E, the fraction of mitotic cells of ZGRF1 deletion was much higher at early phase, espcially at 1 h, and showed no difference at the late time points compared with WT. When cells were treated with nocodazole immediately after irradiation, the WT cells only showed a slight increase of mitotic cells at 1 h time point. Interestingly, the fraction of mitotic cells increased dramatically at 1 h and increased slightly at 2 h time point in ZGRF1 deleted cells. And the accumulation of mitotic cells was ceased at the late time point in both WT and ZGRF1 deleted cells, which was same as Fig. 5E. Based on the above results, we concluded that ZGRF1 acts as an accelerator in the initiation of G2/M checkpoint, but not maintenance.

### ZGRF1 is a new potential target for cancer therapy of both DNA damage drugs and irradiation

**Fig. 6:**
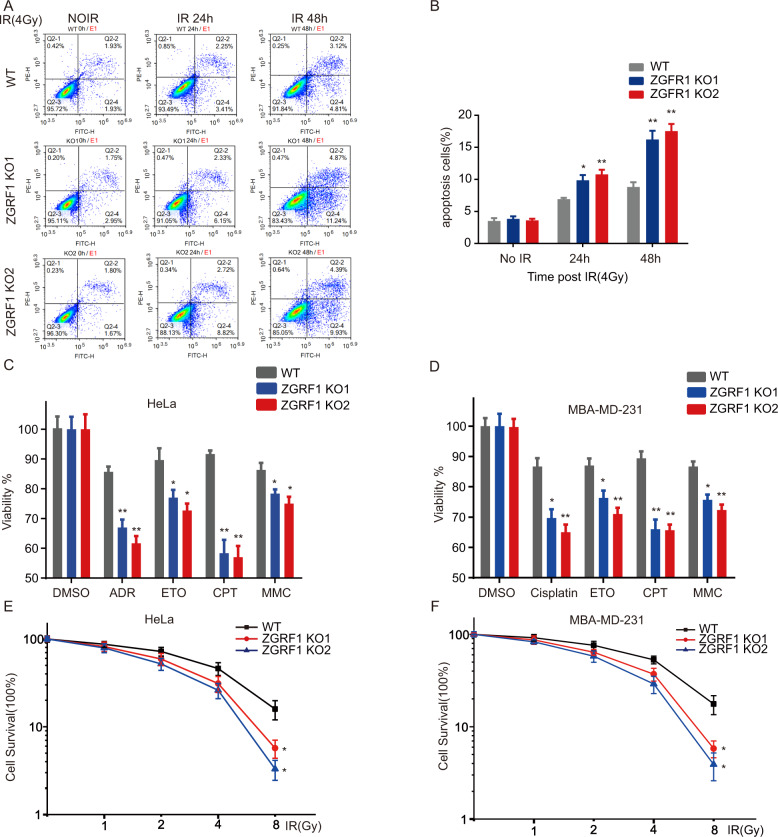
ZGRF1 is a new potential target for cancer therapy of both DNA damage drugs and irradiation. **A** Flow cytometric histograms of apoptosis detection. The WT and ZGRF1 deletion HeLa cells were treated with 4-Gy irradiation. Apoptosis was detected at 24 and 48 h after treatments. **B** Quantification of apoptosis induction. Data are means ± SD from three independent experiments. *P ≤ 0.05; **P ≤ 0.01. **C**, **D** Sensitivity of WT and ZGRF1 deletion HeLa (**C**) and MBA-MD231 (**D**) cells to DNA damage or replication stress–inducing agents was determined by MTS assays. Data are means ± SD from three biological triplicates. **E**, **F** Survival of WT and ZGRF1 deletion HeLa (**E**) and MBA-MD231 (**F**) cells exposed to irradiation treatment. Data are means ± SD from three independent experiments. *P ≤ 0.05; **P ≤ 0.01.

Mitotic cells are hypersensitive to DNA damage, so G2/M checkpoint is important for cell survival in response to DNA damage, otherwise it will produce dicentric chromosomes,aneuploidy and induce cell death [40, 41]. Given ZGRF1 impaired DNA damage repair by disrupting the DNA end resection, and therefore delayed the G2/M DNA damage arrestment.We consider whether ZGRF1 deletion can promote apoptosis of cancer cells after irradiation. We found that the apoptosis percentage of ZGRF1 knock-out HeLa cells increased markedly after treatment with IR (Fig. 6A and B). We also found that ZGRF1 deletion sensitized HeLa (Fig. 6C) and MD231 cells (Fig. 6D) to other DNA-damaging agents, including camptothecin, mitomycin C, and hydroxyurea. The colony formation assay of HeLa cells (Fig. 6E) and MD231 cells (Fig. 6F) confirmed that ZGRF1 deletion cancer cells were extremely sensitive to IR induced DNA damage. Taken together, our data indicates ZGRF1 is a new potential target for cancer therapy of both DNA damage drugs and irradiation.

### ZGRF1 is assciated with tumorigenesis and poor prognosis of cancer therapy

**Fig. 7:**
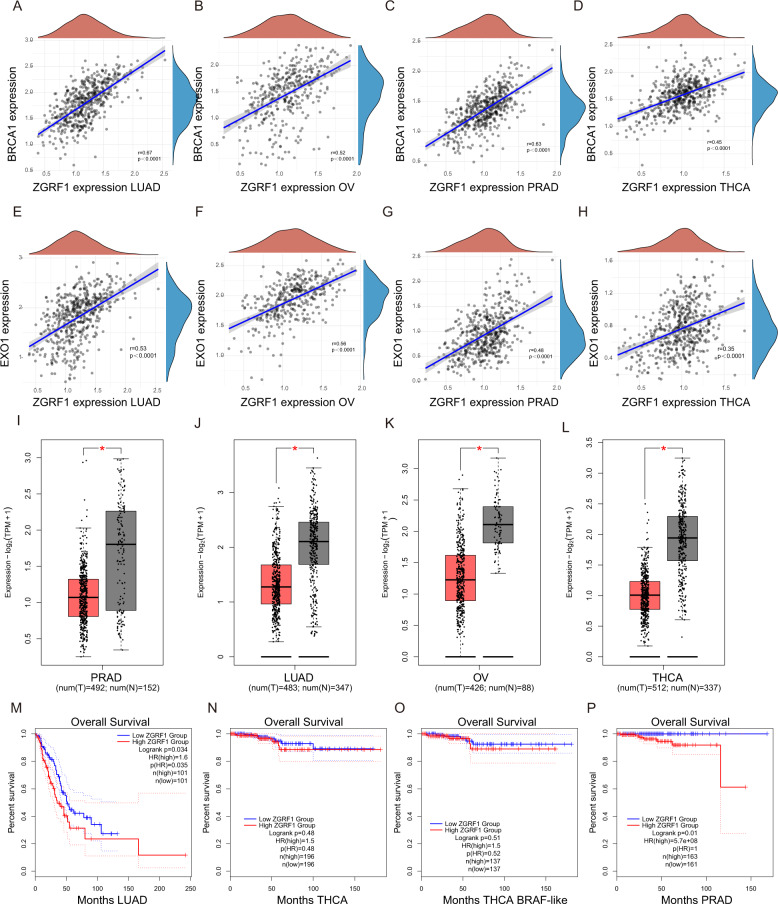
Analyses of correlation between ZGRF1 expression level and clinical cancer features. **A**–**H** Correlations between ZGRF1 expression levels and the mRNA levels of BRCA1 (**A**–**D**) and EXO1 (**E**–**H**) of patients from TCGA datasets. The *r* values and *P* values are from Spearman’s correlation analysis. **I**–**L** ZGRF1 expression in tumor and nontumor tissues of patients from TCGA datasets. **N**–**Q** The overall survival rate analyses in patients from the TCGA dataset.

DNA damage repair genes play vital roles in the maintenance of genome stability. Dysfunction of the cell cycle checkpoint and DNA repair genes are associated with tumorigenesis. We observed that ZGRF1 expression positively correlates with the mRNA levels of BRCA1 and EXO1 in several types of cancer, including lung adenocarcinoma (LUAD), ovarian (OV), prostate adenocarcinoma (PRAD) and tetrahydrocannabinolic acid (THCA) cancers (Fig. 7A–H), suggesting the regulatory of ZGRF1 and BRCA1/EXO1 exists ubiquitous. We next assessed whether ZGRF1 expression levels are correlated with the development of patients with cancers. Indeed, in the aforementioned 4 datasets, the expression of ZGRF1 are significantly lower in tumor tissues than in adjacent nontumor tissues (Fig. 7I–L), indicating that lower expression of ZGRF1 were correlated with tumorigenesis. Kaplan–Meier analysis revealed that lower ZGRF1 levels in tumor tissues were significantly correlated with increased overall survival (OS) rates in LUAD, PRAD, and the BRAF-like type of THCA cancers (Fig. 7M–O).

## Discussion

Following DSB induction, MRN/ATM-CtIP and EXO1/Dna2-dependent DSB end resection results in the formation of ssDNA regions that promotes RPA2 recruitment to damage sites and the following ATR activation and subsequent CHK1 phosphorylation by ATR [42–44]. Consistent with this notion, our data showed that ZGRF1 depletion impairs end resection that significantly reduces the RPA2 foci and the activation of ATR-CHK1 pathway. Similar results have been observed in cells with down regulated EXO1. These results strongly suggest that both ZGRF1 and EXO1 function in the same pathway leading to DSB end resection.Interestingly,unlike BLM, which also promotes EXO1-mediated DNA end resection, depletion does not affect CHK1 phosphorylation and the following G2/M checkpoint, ZGRF1 deletion impairs the initiation of G2/M checkpoint arrestment. This indicates ZGRF1 and EXO1 work in the same pathway. We believe that ZGRF1 plays dual roles in DSB end resection, one is to promote EXO1 nuclease activity,the other is to regulate initial CHK1 activation following DNA replication stress.

The tumor suppressor protein EXO1 also plays an important role in the RPA2 and ATR recruitment and the activation of ATR-CHK1 pathway to induce the cell cycle G2/M checkpoint [45]. However, how this progress is regulated has not been fully understood. Other groups also showed that BLM1 can interact with EXO1 and accelerate EXO1-mediated DNA-end resection.But the other studies also showed that loss of BLM1 does not detectably affect resection,ATR-CHK1 activation, and maintenance of genomic stability or viability [26, 45]. Our data provided novel insights into the molecular basis in promoting DNA-end resection. Here, we report that ZGRF1 interacts with EXO1, which is an executor of DNA end-resection,thus promoting HR. And ZGRF1 deletion also impairs the foci formation of RPA2, and the follow activation of ATR and CHK1 to induce G2/M checkpoint. This suggests ZGRF1 may be the major helicase facilitating EXO1-mediated DNA end resection.

Based on the studies in yeast, it was proposed that DNA end resection is carried out via two steps: the initial end resection by the Mre11 complex and Sae2, and the extended end resection by Sgs1/EXO1 and Dna2 [17, 27]. Previous study showed that EXO1 interacts with the MRE11-RAD50-NBS1 (MRN) complex which is required for EXO1 recruitment to DNA damage sites [45, 46]. BRCA1 also facilitates the recruitment of EXO1. It also has been reported ATM-mediated phosphorylation of CtIP is important for promoting recruitment of BLM and EXO1 to DSBs to initiate HR and the recruitment of BLM and EXO1 to DSBs are dependent on CtIP [14]. This indicates BRCA1 may recruit EXO1 through CtIP. It also has been reported knockdown PCAF(p300/CBP-associated) as a fork-associated protein that promotes fork degradation in BRCA-deficient cells by acetylating H4K8 at stalled replication forks, which recruits EXO1 [47]. Besides,53BP1 knockdown partially restore RPA recruitment in Brca1-null cells which can be negated by additional knockdown of EXO1 [48, 49], indicating EXO1 can also function independently of BRCA1 in resection. We found that MDC1–RNF8–BRCA1 pathway was also essential for ZGRF1 recruitment,Whether ZGRF1 is recruited to DSB depends on its interaction with BRCA1 needs further study. In our study, ZGRF1, which interacts with both BRCA1 and EXO1,deletion reduced the RPA2 foci formation,the marker of DNA end resection,but not EXO1. This indicates BRCA1 not only regulates the recruitment of EXO1 to DNA damage sites,but also promotes EXO1 nuclease activity through ZGRF1.

The helicases is essential in the processing of DNA end resection and homologous recombination. Among them, And-1 promotes the recruitment of CtIP to DNA damage sites, BLM functions in a parallel pathway with EXO1 to promote DSB resection,WRN and RecQ helicase catalyzes unwinding of DNA ends followed by 5′−3′ degradation of the single-strand tails by the Dna2 nuclease [23, 50, 51]. As chemotherapy or radiation frequently introduces DNA damage, helicases that are involved in DNA repair are moving to the forefront of cancer research. Our bioinformation analysis data shows that ZGRF1 expression also positively correlates with the mRNA levels of BRCA1 and EXO1, which are well known for PARPi targets, and higher expression of ZGRF1 predicts poor prognosis of patients in several types of cancer. We also provided experimental evidence that ZGRF1 is also important for EXO1 mediated DNA end resection and the following G2/M checkpoint. ZGRF1 deletion cancer cells sensitivities to CPT, PARPi and irradiation.It indicates the potential of ZGRF1 to be a promising prognostic and efficient medication guidance biomarker for cancer therapy.

In summary, our study demonstrates that ZGRF1 is a critical factor in the maintenance of genome stability through HR-dependent repair of DSBs and EXO1-mediated G2/M checkpoint. Our findings also suggest ZGRF1 is an important helicase to promote EXO1-mediated DNA end resection. We also provide a new sight for the DNA damage-inducing drugs in cancer therapies in targeting ZGRF1 or ZGRF1-dependent processes.

## Materials and methods

### Cell culture and transfection

Hela, MDA-MB231, HEK-293T cells were purchased from the American Type Culture Collection (ATCC). All cells were cultured in DMEM supplemented with 10% fetal bovine serum (FBS) at 37 °C with 5% CO_2_. All transfections were conducted using Lipofectamine 2000 (Invitrogen) according to the manufacturer’s instructions.

### RNA interference target sequences

siRNAs were synthesized by Genepharma. For siRNA transfection, cells were transfected twice at 24 h interval with the indicated siRNA using Lipofectamine®RNAiMAX (Invitrogen) according to the manufacturer’s instructions. The sequences of sgRNAs against human ZGRF1 were:ZGRF1-gRNA1, ATATCCTCTGGCCGATCTCT; ZGRF1-gRNA2, CTGCTACAACTACAGTGTAA; ZGRF1-gRNA3, GATTGGGATTTGAAAGCGG; ZGRF1-gRNA4, ACGCGGGACCTCACAGATG; ZGRF1-gRNA5, GCGGCGGTTTGGCTTAGG. The other siRNA sequences were as follows: 53BP1-siRNA, GAGAGCAGAUGAUCCUUUAdTdT; BRCA1-siRNA, CAGCUACCCUUCCAUCAUAUUdTdT; EXO1-siRNA, CCAAUCUUCUUAAGGGAAATTdTdT; BLM-siRNA, GAGCACAUCUGUAAAUUAAdTdT; RNF8-siRNA, GGAGAUAGCCCAAGGAGAA-dTdT; MDC1-siRNA, GUCUCCCAGAAGACAGUGAdTdT.

### Antibodies and constructs

The following antibodies were used: anti-ZGRF1 (LS-C168135,1:1000 for WB,LSBio), anti-BRCA1 (D-9, dilution:1:100 for IF, Santa Cruz) and (C-20,1:200 for WB and IP, Santa Cruz), anti-EXO1 (ab95012,1:1000 forWB, Abcam), anti-BLM (ab2179, 1:1000 for WB, Abcam), anti-CHK1 pS317 (2344 S, 1:500 for WB, Cell Signaling), anti-CHK1 (sc-7898, 1:1000 for WB, Santa Cruz), anti-CHK2 pT68 (GTX61178, 1:1000 for WB, GeneTex), anti-CHK2 (2662,1:1000 for WB, Cell Signaling Technology), anti-RPA2 pS4/S8 (A300-245A, 1:1000 for WB, Bethyl Laboratories), anti-RPA2 (ab76420; 1:500 for IF; Abcam), anti-γH2AX (05-636; 1:1000 for WB and 1:500 for IF; Millipore)

### Clonogenic survival assay

WT and ZGRF1 knock-out HeLa cells and MD231 cells were plated in 60 mm dishes. Six hours later, cells were treated with olaparib or irradiation with indicated doses. Cells were incubated in 4 ml medium and cultured for 2 weeks to allow colony formation. Cells were stained with 0.5% crystal violet in phosphate-buffered saline (PBS) with 20% methanol, and colonies with >50 cells were counted.

### DNA repair assay

HR and NHEJ assays were used to determine the HR and NHEJ repair efficiency. The DNA repair assays were performed as previously described [52]. Briefly, HEK293 cells integrated with direct repeat GFP (DR-GFP) or EJ5-GFP reporters were infected with the indicated plasmid or siRNA randomly. Then, cells were transfected with I-Scel and p-cherry expression vector. DOX was added to induce I-SecI expression. Forty-eight hours after DOX was added, the percentage of GFP- or RFP-positive cells was analyzed by FACS. HR and NHEJ efficiency were presented as the percentage of GFP- and RFP-positive cells.Repair frequencies presented are means ± SD of at least three independent experiments.

### Cell viability assay

WT and ZGRF1 knock-out HeLa cells and MD231 cells were plated onto 96 well plates (2000 cells per well) and, 6 h later, treated with MMC, CPT, ETO, ADR, Cisplatin, or PARPi as indicated.Two days later, the viability of the cells was determined using the CellTiter-Blue reagent (Promega). During the experiment and assessing the outcome, the investigator was blinded to the cell lines and which drugs were used. Data were presented as means ± SD of at least three independent experiments.

### Immunofluorescence

Cells were cultured on coverslips and treated with 4 Gy of IR. The cells were washed three times with ice-cold PBS 1 h after IR and then incubated with 4% paraformaldehyde at room temperature for 15 min. The cells were subsequently permeabilized with PBS containing 0.5% Triton X-100 at room temperature for 10 min and blocked with 10% FBS in PBS at room temperature for 1 h. Then, cells were incubated for 1 h with primary antibodies at room temperature. Cells were subsequently washed three times with PBS and then incubated with secondary antibodies. Then, 4’,6-diamidino-2-phenylindole (DAPI) staining was performed. Slides were imaged using a Zeiss LSM800 fluorescence microscope.

### Co-IP and Western blotting

For Co-IP assay, cells were lysed in NETN buffer [100 mM NaCl, 20 mM tris-Cl (pH 8.0), 0.5 mM EDTA, 0.5% (v/v) NP-40, 1× cocktail protease inhibitor] and maintained under constant agitation for 30 min at 4 °C followed by centrifugation for 10 min at 4 °C. Following centrifugation, the supernatant was treated with 50 μg/ml DNAase to remove chromatin.Then, the cell lysis was incubated with indicated antibodies for 6 h at 4 °C and washed three times with NETN buffer. The samples were separated by SDS-polyacrylamide gel electrophoresis (SDS-PAGE) and detected with indicated antibodies.For Western blot, cells were lysed in NETN buffer and maintained under constant agitation for 30 min at 4 °C. Samples were separated by SDS-PAGE and detected with indicated antibodies.

### Mass spectrometry

HeLa cells were harvested 1 h after 4 Gy irradiation treatment and immunoprecipitated as described in the Co-IP procedure. Then samples were separated by SDS-PAGE and stained with Coomasssie bluede.Mass spectrometry analysis of protein–protein ineraction was performed by PTM Biolabs Inc (Hangzhou,China).

### Flow cytometry

During the experiment and assessing the outcome, the investigator was blinded to the cell lines and treatment. Cells were trypsinized and washed with ice-cold PBS. For analysis of the cell cycle, cells were fixed in ice-cold 70% ethanol overnight at −20 °C and then centrifuged at 1000 rpm at 4 °C for 5 min,and pellets were suspended in 500 ml of PBS containing propidium iodide (100 mg/ml) and ribonuclease (10 mg/ml) for 30 min at room temperature. For analysis of apoptosis, the Annexin V-FITC Apoptosis Detection Kit (Beyotime) was used according to the manufacturer’s instruction. Fluorescence-activated cell sorting (FACS) analysis was performed by flow cytometry, and the percentage of apoptotic cells and cells in the G0-G1, S, and G2-M phases of the cell cycle was analyzed by ModFit (version 2.0) software.

Monitoring of mitotic H3 phosphorylation was carried out as follows.Cells harvested by trypsinization were washed and fixed for 10 min at 37^o^C in PBS/1% methanol-free formaldehyde.After cooling the cells were permeabilized by addition of methanol to a final concentration of 90%. Cells were stained with Alexa 488-conjugated anti-Phospho(Ser10)H3 (Cell Signaling Technology) according to the manufacturer’s instructions.Cells were then washed with PBS/0.5% BSA and treated with RNase A and propidium iodide. H3 phosphorylation was analyzed using a FACS Calibur (BD Biosciences). FlowJo software (Treestar, Inc.) was used to quantitate levels of mitotic H3 phosphorylation.

### Statistical analysis

The dataset used to comprise mRNA-seq data was from TCGA tumors (see TCGA Data Portal at https://tcga-data.nci.nih.gov/tcga/.For analyses of correlation between the ZGRF1 expression levels and clinical features,Spearman’s tests were used. Survival curves were calculated using Kaplan–Meier method,and the significance was determined by log-rank test. A *p*-value of less than 0.05 was considered statistically significant.

The experimental results are expressed as the mean ± standard deviation and were calculated from quantitative data obtained from three replicate experiments. Statistical analysis was performed using one-way analysis of variance in SPSS v18.0 software. The significance of the differences between two groups were determined using LSD *t*-test. The *p*-values ≤ 0.05 were considered significant.

## Supplementary information

author contribution

## Data Availability

All data needed to evaluate the conclusions in the paper are present in the paper and has been repeated for at least 3 times in the laboratory. Additional data related to this paper may be requested from the authors. The data and materials used or analyzed during the current study are available from the corresponding author on reasonable request.
