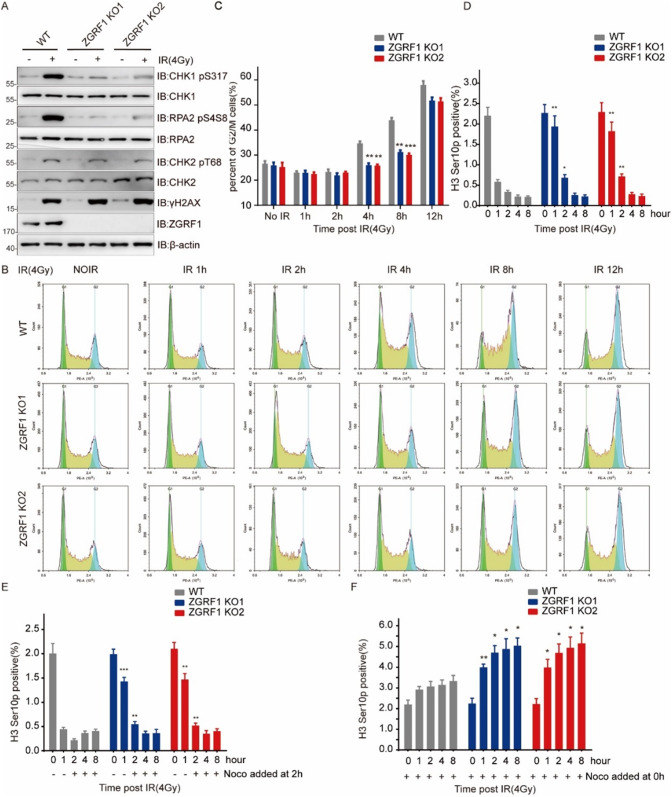# Correction to: ZGRF1 promotes end resection of DNA homologous recombination via forming complex with BRCA1/EXO1

**DOI:** 10.1038/s41420-022-01248-2

**Published:** 2022-11-18

**Authors:** Shuang Yan, Man Song, Jie Ping, Shu-ting Lai, Xiao-yu Cao, Chen-Jun Bai, Da-Fei Xie, Hua Guan, Shan-shan Gao, Ping-Kun Zhou

**Affiliations:** 1 Institute for Environmental Medicine and Radiation Hygiene, School of Public Health, University of South China, Hengyang, Hunan Province People’s Republic of China; 2 Department of Radiation Biology, Beijing Key Laboratory for Radiobiology, Beijing Institute of Radiation Medicine, Beijing, People’s Republic of China; 3 State Key Laboratory of Proteomics, National Center for Protein Sciences, Beijing Institute of Radiation Medicine, Beijing, People’s Republic of China; 4 College of Life Sciences, Hebei University, Baoding, He Bei Province People’s Republic of China

**Keywords:** Targeted therapies, DNA

Correction to: *Cell Death Discovery* (2021) 7:260 10.1038/s41420-021-00633-7, published online 22 September 2021

There is an error in Figure 5 of the originally published article, where a wrong flow cytometric histogram was unfortunately uploaded in placed of ZGFR1 KO2 panel- IR 12h in Figure 5B. The correct Figure 5 can be found below. The original article has been corrected.