# Supplementary material for: ZGRF1 promotes end resection of DNA homologous recombination via forming complex with BRCA1/EXO1
Source: Cell Death Discov. 2021 Sep 22;7:260. doi: 10.1038/s41420-021-00633-7 (PMC8458317; doi:10.1038/s41420-021-00633-7)
Supplement: Supplementary file 1 — author contribution [file 41420_2021_633_MOESM1_ESM.docx]

Author contributions: S.-S.G. and S.Y. performed most of the experiments. J.P. did the bioinformation analysis of ZGRF1. S.-T.L., X.-Y.C.,L.G.,J.J.,M.S.,C.-J.B.,H.G.,D.-F. Xie assisted with the experiment and provided technical help. P.-K.Z. and S.-S.G. contributed study concept and critical design. S.-S.G. and P.-K.Z. conceived the project and analyzed the data. S.Y, S.-S.G. drafted the initial manuscript. P.-K.Z. critically reviewed and revised the final manuscript.
